# Thermally-Induced Actuations of Stimuli-Responsive, Bicompartmental Nanofibers for Decoupled Drug Release

**DOI:** 10.3389/fchem.2019.00073

**Published:** 2019-02-19

**Authors:** Chan Woo Jung, Jae Sang Lee, Ghulam Jalani, Eun Young Hwang, Dong Woo Lim

**Affiliations:** Department of Bionano Engineering and Bionanotechnology, College of Engineering Sciences, Hanyang University, Ansan, South Korea

**Keywords:** thermal responsiveness, bicompartmental nanofibers, actuations, electrohydrodynamic co-jetting, decoupled drug release

## Abstract

Stimuli-responsive anisotropic microstructures and nanostructures with different physicochemical properties in discrete compartments, have been developed as advanced materials for drug delivery systems, tissue engineering, regenerative medicine, and biosensing applications. Moreover, their stimuli-triggered actuations would be of great interest for the introduction of the functionality of drug delivery reservoirs and tissue engineering scaffolds. In this study, stimuli-responsive bicompartmental nanofibers (BCNFs), with completely different polymer compositions, were prepared through electrohydrodynamic co-jetting with side-by-side needle geometry. One compartment with thermo-responsiveness was composed of methacrylated poly(N-isopropylacrylamide-co-allylamine hydrochloride) (poly(NIPAM-co-AAh)), while the counter compartment was made of poly(ethylene glycol) dimethacrylates (PEGDMA). Both methacrylated poly(NIPAM-co-AAh) and PEGDMA in distinct compartments were chemically crosslinked in a solid phase by UV irradiation and swelled under aqueous conditions, because of the hydrophilicity of both poly(NIPAM-co-AAh) and PEGDMA. As the temperature increased, BCNFs maintained a clear interface between compartments and showed thermally-induced actuation at the nanoscale due to the collapsed poly(NIPAM-co-AAh) compartment under the PEGDMA compartment of identical dimensions. Different model drugs, bovine serum albumin, and dexamethasone phosphate were alternately loaded into each compartment and released at different rates depending on the temperature and molecular weight of the drugs. These BCNFs, as intelligent nanomaterials, have great potential as tissue engineering scaffolds and multi-modal drug delivery reservoirs with stimuli-triggered actuation and decoupled drug release.

## Introduction

Development of stimuli-responsive, multi-compartmentalized microstructures or nanostructures in the form of particles, cylinders, and fibers has increased interest in a variety of industrial and biomedical applications because they have different physicochemical, optical, and electromagnetic properties and environmental sensitivity in each compartment (Bhaskar et al., 2009; Rahmani and Lahann, 2014). These structures have been widely applied to electronic paper devices, switchable displays, colloidal stabilizers at an interface, self-propelled motors, spontaneous formation of complex structures, multiplexed optical biosensors, multi-modal drug delivery systems, tissue engineering scaffolds, and filamentous actuators for soft robotics (Kaewsaneha et al., 2013; Jung et al., 2014; Pang et al., 2014; Stoychev and Ionov, 2016; Zhou et al., 2016). Specifically, multi-compartmental polymer microcylinders and nanofibers with compositional anisotropy showed controlled shape reconfigurations and reversible anisotropic actuations, depending on environmental stimuli including ultrasound, solvent exchange, temperature, pH, and ionic strength (Lendlein et al., 2001; Chen et al., 2010; Lee et al., 2012; Liu et al., 2012; Qi et al., 2016). These stimuli-triggered, swelling-deswelling properties of each compartment occurred to different degrees via volume change, which was largely affected by hydrophilicity-hydrophobicity and conformational changes of the polymers (Okeyoshi et al., 2008; Rockwood et al., 2008; Okuzaki et al., 2011; Saha et al., 2012; Stoychev and Ionov, 2016; Hessberger et al., 2018).

A number of original platform technologies for synthesis of multi-compartmentalized structures at micro- or nanoscale have been devised based on composition and surface anisotropy: electrohydrodynamic (EHD) co-jetting, microfluidics, flow-focusing lithography, template-assisted polymerization, differential solvent evaporation, spinning disks, selective crystallization, and deposition, partial masking, Pickering emulsion, and self-assembly (Paunov, 2003; Bong et al., 2009; Pardhy and Budhlall, 2010; Du and O'reilly, 2011; Jung et al., 2014; Guignard and Lattuada, 2015; Nisisako, 2016). In particular, EHD co-jetting enables different polymer solutions in miscible aqueous or organic solvent with side-by-side needle geometry to have equilibrated laminar flow and ejection from the vertex of the multi-phasic Taylor cone in a thin-jet stream, induced by charge-charge repulsion under high electrical potential (Barrero et al., 1998; Hogan Jr et al., 2007). This has significant advantages over the other preparation methods: (1) simple scale-up to prepare micro- or nanosized, multi-compartmentalized structures with completely different materials in each compartment, (2) easy control over the three-dimensional geometry and aspect ratio of each compartment, (3) facile introduction of inorganic nanomaterials in a colloidal state; biomacromolecules such as DNAs, RNAs, and proteins; and therapeutic drugs within each compartment, and (4) versatility of spatio-selective modification by immobilization of different biological ligands onto the surface of the respective compartment for active drug targeting (Bhaskar et al., 2008, 2011; Jaworek and Sobczyk, 2008). As reported, gold nanocrystals, titanium dioxides, magnetites, and small interfering RNAs were homogeneously encapsulated into distinct compartments for theranosis, and the spatioselectively immobilized biological ligands on the bicompartmental nanofibers or nanoparticles mediated directional cell growth and stereo-specific binding of each compartment on the substrate via noncovalent molecular interactions for tissue engineering and multiplexed biosensing (Mandal et al., 2009; Hwang et al., 2010; Lim et al., 2010; Misra et al., 2012; Jung et al., 2014). In addition, chemically controlled bending and reversible shape shifting of multi-compartmental microcylinders induced by different volume transitions were reported for microactuator-based biomedical applications (Saha et al., 2012). Furthermore, anisotropic nanofiber scaffolds composed of physically crosslinked poly(N-isopropylacrylamide-co-stearyl acrylate) (poly(NIPAM-*co*-SA)) compartments with thermal responsiveness and chemically crosslinked poly(ethylene glycol) (PEG) compartments showed thermally-induced shape changes due to the collapsed poly(NIPAM-*co*-SA) compartment under aqueous conditions (Jalani et al., 2014). However, there was no clear reversible transition from fully stretched to coiled shapes, potentially due to the physically crosslinked poly(NIPAM-*co*-SA) networks formed by hydrophobic interactions between SA moieties, limiting their applications such as microactuators, temporally multi-modal drug delivery reservoirs, and shape-morphing hydrogel scaffolds (Browne and Pandit, 2014; Jeon et al., 2017; LöWenberg et al., 2017).

In this study, thermally-responsive and chemically-crosslinked bicompartmental nanofibers (BCNFs) with complete compositional anisotropy were prepared by EHD co-jetting with side-by-side needle geometry and UV irradiation. The BCNFs are composed of methacrylated poly(N-isopropylacrylamide-co-allylamine hydrochloride) (poly(NIPAM-co-AAh)) with thermal responsiveness and poly(ethylene glycol) dimethacrylates (PEGDMA) in separate compartments. Both the methacrylated poly(NIPAM-co-AAh) and PEGDMA in the distinct compartments were chemically crosslinked in a solid phase by UV irradiation and stabilized under aqueous conditions. We hypothesized that these BCNFs would be swollen under aqueous conditions because of hydration of chemically crosslinked poly(NIPAM-co-AAh) and PEGDMA in the two compartments, and they would show a thermally-induced transition from extended to coiled shapes due to thermally-induced collapse of the poly(NIPAM-co-AAh) compartment. The BCNFs maintained a distinct interface between the poly(NIPAM-co-AAh) and PEGDMA compartments in both dry and swollen states under aqueous conditions. As temperature increased, the BCNFs showed thermally-induced actuations because the poly(NIPAM-co-AAh) compartment collapsed due to aggregated poly(NIPAM-co-AAh) under the identical dimensions of the PEGDMA compartment. However, no thermally-induced coiling was observed in single compartmental nanofibers (SCNFs) of the methacrylated poly(NIPAM-co-AAh) and PEGDMA used as controls. As a proof of concept, when different model drugs, bovine serum albumin (BSA) as a biomacromolecule, and dexamethasone phosphate as a small molecule were alternately loaded into each compartment, they were released at different rates depending on temperature and molecular weights (MWs) of the drugs. These BCNFs have potential as tissue engineering scaffolds and multi-modal drug delivery reservoirs with stimuli-triggered actuation at the nanoscale and decoupled drug release.

## Experimental Part

### Materials

*N*-isopropylacrylamide (NIPAM; 97 %), allylamine, hydrochloric acid, tert-butyl alcohol, and diethyl phosphite were obtained from Sigma Aldrich (St Louis, MO, USA). The 2,2'-azobis(2-methylpropionitrile) (AIBN; 98 %) obtained from Acros Organics (Morris Plains, NJ, USA) was purified by recrystallization in methanol. Poly(ethylene glycol) (MW: 20,000 g/mol), dichloromethane, N,N-dimethylformamide (DMF), methacrylic anhydride, triethylamine, 2-hydroxy-2-methyl-propiophenone (97%) as a photoinitiator, fluorescein diacetate, Nile red, ethanol, 2,2,2-trifluoroethanol (TFE), dexamethasone 21-phosphate (DMP) disodium salt, BSA, and phosphate buffered saline (PBS) were obtained from Sigma Aldrich. Deionized water was purified by Milli-Q (Millipore Water Purification Systems, Bedford, MA, USA) and used throughout all experiments.

### Synthesis and Characterization of Methacrylated Poly(NIPAM-co-AAh) and PEGDMA

In a typical experiment, 6.33 M hydrochloric acid (35.0 v/v %) was added to 10.86 M allylamine under stirring at 4°C to prepare allylamine hydrochloride. Next, water and an excess of hydrogen chloride were removed using a rotary evaporator at 60°C. NIPAM was dissolved in DMF at room temperature and then recrystallized below 10°C to remove any impurities. Twenty grams of NIPAM were dissolved in 200 mL of DMF in a flask at a final concentration of 10.0 w/v%, and the NIPAM solution was then transferred to a refrigerator at 4°C for recrystallization. The solution containing recrystallized NIPAM was filtered through filter paper (Whatman® qualitative filter paper, grade 1; Whatman plc, Maidstone, UK) via an aspirator assembly (EYELA 1000S; EYELA, Bohemia, NY, USA). The recrystallized NIPAM was then dried to remove residual organic solvent. Poly(NIPAM-co-AAh) was synthesized by free radical polymerization of NIPAM and allylamine hydrochloride as the monomers, as reported previously (Jaber and Schlenoff, 2005). Solution A, containing 0.09 M NIPAM and 0.03 M allylamine hydrochloride dissolved in tert-butyl alcohol, was added to 5.8 mM diethyl phosphite. Solution B was prepared by adding AIBN (0.2 mol %) to 2.5 mL tert-butyl alcohol. Next, solution A was added to a 250-mL three-necked round-bottom flask and bubbled with nitrogen for 10 min. We placed solution A in a water bath and heated it to 83°C; then, solution B was quickly added to the flask to initiate radical polymerization. The reaction was carried out for 24 to 36 h under constant stirring. Once polymerization was complete, the solution temperature was lowered to 4°C, and the polymer was precipitated by adding ethyl acetate, which was followed by vacuum drying at 80°C for 18 h to remove remaining organic solvent. To introduce methacrylic moieties to poly(NIPAM-co-AAh) for UV-mediated chemical crosslinking in the solid phase, 1 mM methacrylic anhydride in water was added to poly(NIPAM-co-AAh) solution at 4°C for 8 h, dialyzed against water at 4°C for 48 h, and freeze-dried by a lyophilizer (MCFD8508; Ilshin Lab Co., Ltd., Gyeonggi-do, Korea).

PEGDMA was prepared from the reaction of methacrylic anhydride (MA) and PEG (MW: 20,000 g/mol) at room temperature, as reported previously (Lin-Gibson et al., 2004). PEG, MA, and triethylamine were dissolved in 15 mL dichloromethane at a feed molar ratio of 0.025:0.22:0.01, and the solution was stirred at room temperature for 4 days. Finally, PEGDMA was precipitated by adding an excess amount of diethyl ether, then filtering the mixture and drying it in a vacuum oven to remove the unreacted monomers.

Poly(NIPAM-co-AAh) and methacrylated poly(NIPAM-co-AAh) were analyzed by GPC to determine their average molar masses and molar mass distributions. The GPC analysis was performed by a Waters-515 HPLC pump system with a 2,410 refractive index (RI) detector. A Shodex GPC column, KF-803 (Shodex GPC system-21, Showa Denko Co., Tokyo, Japan) and polystyrenes in the MW range of 820 to 1,070,000 g/mol were used to create a standard curve. Tetrahydrofuran as the mobile phase was used at a flow rate of 1.0 mL/min. The chemical structures of poly(NIPAM-co-AAh), methacrylated poly(NIPAM-co-AAh) and PEGDMA in D_2_O as a solvent were confirmed from the ^1^H nuclear magnetic resonance (NMR) analysis using a Bruker AVANCE III 400 MHz spectrometer (Bruker BioSpin AG, Fallanden, Switzerland). The apparent molar ratio of NIPAM and AAh of poly(NIPAM-co-AAh) and incorporation efficiency of methacrylic groups into allylamine residues of poly(NIPAM-co-AAh) or hydroxyl groups of PEGs were determined by calculating the relative areas under the curves of the corresponding peaks in the ^1^H NMR spectrum. In addition, to characterize the thermal responsiveness of the poly(NIPAM-co-AAh) and methacrylated poly(NIPAM-co-AAh), the absorbance of the polymer solution in water at 0.5 and 2.0 w/v % as a function of temperature in the range of 25–50°C was measured at 360 nm using a UV-visible (UV-Vis) spectrometer (Cary-100 Bio, Varian Biotech, Palo Alto, CA, USA) with a Peltier thermostat for temperature control at a heating rate of 1°C /min as previously described (Jalani et al., 2014).

### Preparation and Stabilization of BCNFs by EHD Co-jetting

Two polymer solutions for EHD co-jetting were separately prepared in a solvent mixture of ethanol and TFE at a 3:1 volume ratio. Fluorescein diacetate and Nile red as hydrophobic fluorescence dyes were separately added to each polymer solution to characterize the biphasic Taylor cone during EHD co-jetting, an interface between two compartments, and the degree of anisotropy using the different emission spectra of the two dyes. One polymer solution contained 0.30 g methacrylated poly(NIPAM-co-AAh) and 0.5 mg fluorescein at final concentrations of 30.0 and 0.05 w/v%, respectively. The other polymer solution was comprised of 0.24 g PEGDMA and 0.5 mg Nile red at final concentrations of 24.0 and 0.05 w/v%, respectively. In addition, 2-hydroxy-2-methyl-propiophenone as a photoinitiator was added to each polymer solution at a final concentration of 1.0 v/v% to chemically crosslink the BCNFs via UV irradiation. The two different polymer solutions were separately loaded into each 1.0-mL syringe (NORM-JECT Luer; Henke-Sass, Wolf, Tuttlingen, Germany) connected to a dual channel needle (Fibrijet SA-3610, Micromedics, Inc., Eagan, MN, USA) with a side-by-side geometry. An applicator (Fibrijet; Micromedics, Inc.) to fix two syringes and a micro syringe pump (KD Scientific, Holliston, MA, USA) were used for precise and simultaneous control over the flow rates of the two polymer solutions. A power supply (NanoNC Co., Ltd., Seoul, Korea) was introduced to apply a high voltage between the dual needles and an aluminum foil with a thickness of 0.018 mm (Fisherbrand, Thermo Fisher Scientific, Hillsboro, OR, USA). The cathode was connected to the dual needles, while the anode was connected to the foil with a vertical distance in the range of 10–15 cm. Both high DC electrical potential in the range of 9–10 kV and flow rate in the range of 0.40–0.50 mL/h were maintained during EHD co-jetting. A digital camera (D-90, Nikon, Tokyo, Japan) with high resolution was used to check the biphasic Taylor cone, jet stream, and jet breakup and collect photographic images during EHD co-jetting. As controls, SCNFs composed of either methacrylated poly(NIPAM-co-AAh) or PEGDMA were separately prepared as described above. All compartments of the BCNFs and SCNFs were chemically crosslinked in the solid state by UV irradiation for 120 min using a UV lamp (Omnicure-1500A, Lumen Dynamics Group Inc., Toronto, Canada) operating at a power of 60 mW/cm^2^. The stabilized BCNFs and SCNFs were confirmed in dry and swollen states by fluorescence microscopy and confocal laser scanning microscopy (CLSM).

### Characterization of BCNFs and SCNFs

Both BCNFs and SCNFs were characterized in the dry state using an IX81 inverted phase contrast fluorescence microscope (Olympus, Tokyo, Japan) in bright field and fluorescence modes. In particular, the degree of compartmentalization of the BCNFs was confirmed using the TCS SL CLSM (Leica, Wetzlar, Germany) equipped with He/Ne and argon lasers. To avoid any overlap of emission signals of the two fluorescence dyes, the wavelength ranges of the spectral emissions of fluorescein and Nile red were optimized to 543–579 and 592–647 nm, respectively. The chemically stabilized BCNFs were homogeneously dispersed in PBS at pH 7.4, sonicated using an ultrasonicator (VC 505, Sonics and Materials, Inc., Newtown, CT, USA), and then imaged to characterize chemical stability, swelling properties, maintenance of the interface between two compartments, and compartmentalization degree of the BCNFs under aqueous conditions. A temperature-controlled stage (Tempcontroller 37–2 digital, PeCon, Erbach, Germany) was applied to the CLSM to observe any thermally-induced shape change. The temperature was controlled in the range of 5–45°C, and any shape changes induced by thermal triggers were observed in real time and recorded as fluorescence images by CLSM. Identical conditions for CLSM imaging of the BCNFs were used to image the SCNFs composed of methacrylated poly(NIPAM-co-AAh) or PEGDMA as controls. In addition, scanning electron microscopy (SEM) was used to characterize the diameters, diameter distributions, and surface morphologies of BCNFs and SCNFs in the dry state. They were coated with platinum using a K575X Turbo Sputter Coater (Emitech, East Sussex, UK) to introduce a conductive layer and then imaged by SEM (S-4800, Hitachi, Tokyo, Japan) operated at an accelerating voltage of 0.5–30 kV.

### Preparation of Dual Drug-Loaded BCNFs and *in vitro* Release

DMP and BSA were introduced separately into each compartment of the BCNFs as small molecular and biomacromolecular drugs via EHD co-jetting. In general, two polymer solutions having different model drugs were separately prepared. The first polymer solution contained 0.36 g methacrylated poly(NIPAM-co-AAh) and 2.0 mg of DMP or BSA in 1.0 mL of a solvent mixture of TFE and ethanol at a 1:3 volume ratio. In contrast, the other polymer solution was comprised of 0.24 g PEGDMA and 2.0 mg of BSA or DMP in 1.0 ml of the identical solvent mixture. As mentioned above, two different dual drug-loaded BCNFs were prepared via EHD co-jetting: (1) the BCNFs composed of a DMP-loaded, methacrylated poly(NIPAM-co-AAh) compartment and a BSA-loaded PEGDMA compartment and (2) the other BCNFs with a BSA-loaded methacrylated poly(NIPAM-co-AAh) compartment and a DMP-loaded PEGDMA one.

Ten milligrams each of two different dual drug-loaded BCNFs were suspended in 1 mL of PBS at pH 7.4 to study the drug release kinetics from each compartment to observe temperature-controlled drug release via volume change. The BCNF solutions in a shaker operating at 100 rpm were incubated at 4 or 37°C. The *in vitro* drug release of five different batches was measured to study batch-to-batch variability. Concentrations of DMP and BSA in aliquots of the BCNF solutions at predetermined times were determined with a UV-Vis spectrophotometer by measuring the optical absorbance at 242 nm for DMP and at 278 nm for BSA.

## Results and Discussion

**Figure 1:**
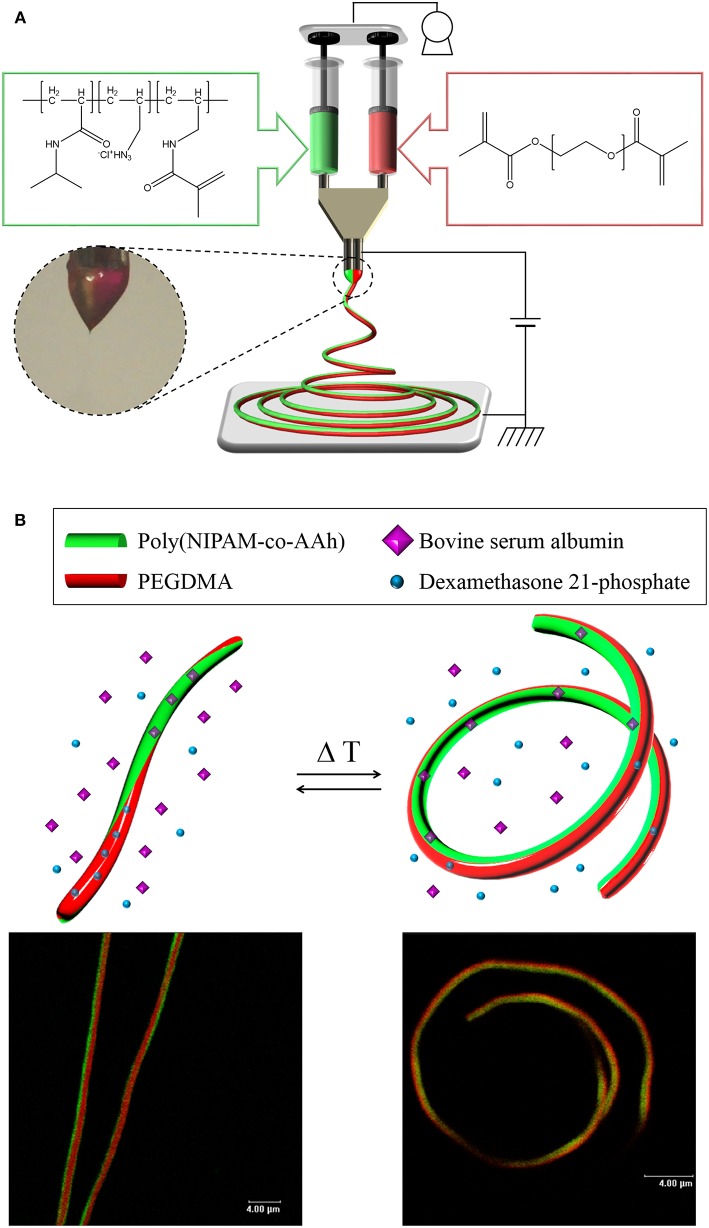
**(A)** Schematic of electrohydrodynamic co-jetting of different polymer solutions with side-by-side needle geometry, methacrylated poly(NIPAM-co-AAh) with fluorescein diacetate, and PEGDMA with Nile red, in a mixture of ethanol and TFE at a 3:1 volume ratio for synthesis of stimuli-responsive, chemically crosslinked BCNFs. **(B)** Cartoons and CLSM images of temperature-triggered, reversible nanoscale actuation of the chemically crosslinked BCNFs, where the poly(NIPAM-co-AAh) compartment was thermally responsive but the PEGDMA one was not. Scale bars in the fluorescence images are 4.0 μm.

Our hypothesis in this study was that from these stimuli-responsive, chemically crosslinked BCNFs composed of a thermally responsive poly(NIPAM-co-AAh) compartment and a non-thermally responsive PEGDMA, one could clearly show reversible, thermally-induced shape change from fully extended to coiled nanostructures, potentially due to the different volume transitions of each compartment, which would result from the thermally triggered collapse of the chemically crosslinked poly(NIPAM-co-AAh) networks and unchanged volumes of the PEGDMA networks in an aqueous environment. As shown in Figure 1A, EHD co-jetting of completely different polymer solutions, methacrylated poly(NIPAM-co-AAh) with fluorescein diacetate and PEGDMA with Nile red, in a mixture of ethanol and TFE at a 3:1 volume ratio was performed with side-by-side needle geometry. Two fluorescent dyes, fluorescein and Nile red, were introduced into the polymer solutions to check the biphasic Taylor cone during EHD co-jetting and to characterize both the interface and the degree of compartmentalization using their different emission spectra according to CLSM. In addition, 1.0 v/v% 2-hydroxy-2-methyl-propiophenone as a photoinitiator was added to both polymer solutions to chemically crosslink polymeric networks of both compartments in the solid phase via UV irradiation. Although the two jetting solutions contained completely different polymer compositions, 15,000 g/mol for methacrylated poly(NIPAM-co-AAh) and 20,000 g/mol for PEGDMA as the molar masses were very close, and 24.0–30.0 w/v% polymer concentrations in the 3:1 ethanol and TFE mixture in the two jetting solutions, were used to balance the viscosities, surface tensions, and conductivities of the two polymer solutions. When the distance between electrodes was optimized in the range of 10–15 cm, the uniform cone-jet mode of the biphasic Taylor cone was maintained under 9–10 kV of electrical potential and 0.40–0.50 mL/h of constant laminar flow during the EHD co-jetting, as shown in the photographic image. The resulting BCNFs were photo-crosslinked by UV irradiation in the solid state for stabilization under aqueous conditions. Figure 1B represents both cartoons and CLSM images of the temperature-triggered, reversible nanoscale actuation of the chemically crosslinked BCNFs, where the poly(NIPAM-co-AAh) compartment was thermally responsive, while the PEGDMA one was not. As reported in bi-gels having anisotropically distributed poly(NIPAM) with lower critical solution temperature (LCST) behavior (Hu et al., 1995), the poly(NIPAM-co-AAh) compartment at 40°C (above LCST) showed a dramatic volume decrease compared to its volume at 10°C (below LCST) due to contraction of poly(NIPAM-co-AAh) chains in the networks, while no significant volume change below or above LCST occurred in the PEGDMA compartment. In particular, when DMP and BSA as small molecular and biomacromolecular model drugs were separately encapsulated within these BCNFs, the *in vitro* release rates of the model drugs in PBS were largely affected by temperature-induced volume changes of the poly(NIPAM-co-AAh) and PEGDMA compartments.

### Polymer Synthesis and Characterization

**Figure 2:**
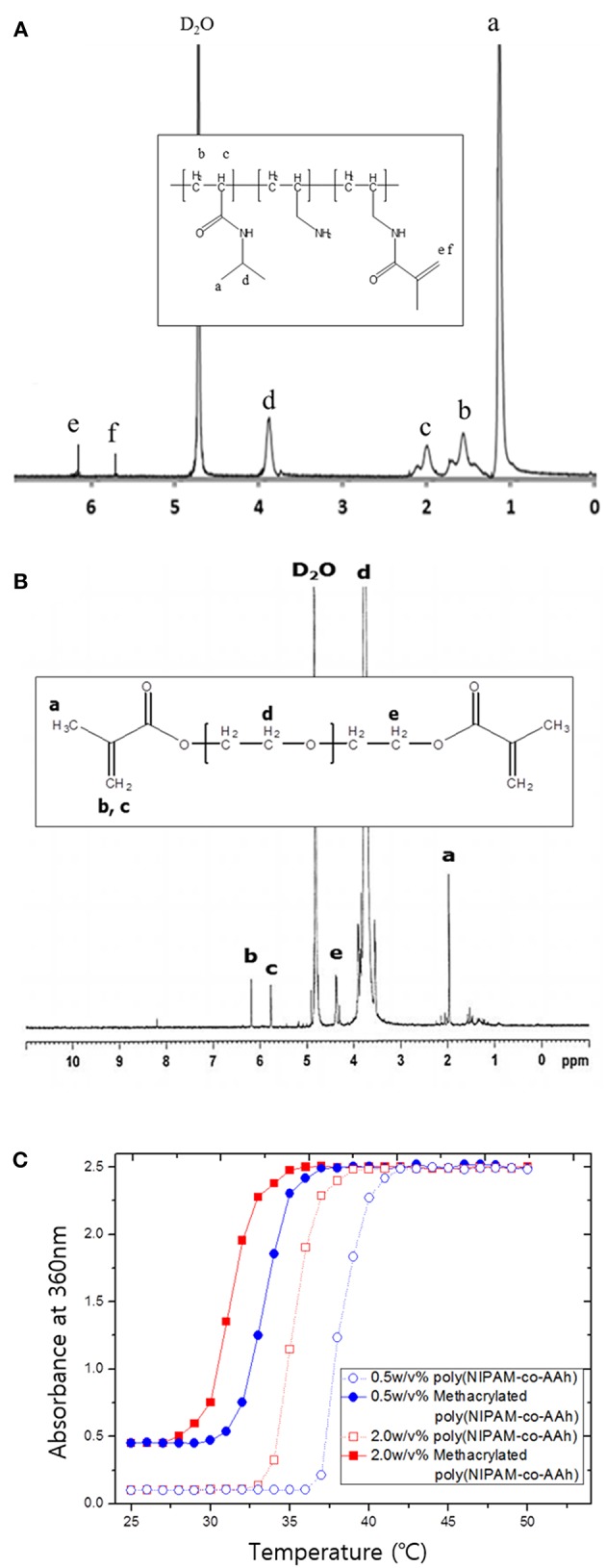
^1^H nuclear magnetic resonance spectra of **(A)** methacrylated poly(NIPAM-co-AAh) and **(B)** PEGDMA. **(C)** UV-Vis absorption spectra of poly(NIPAM-co-AAh) and methacrylated poly(NIPAM-co-AAh) at different polymer concentrations in the range of 20–60°C.

Poly(NIPAM-co-AAh) was synthesized at 83°C by free radical polymerization of NIPAM and AAh as monomers in the presence of AIBN as a thermal initiator. As determined by GPC analysis, the number average molar mass was 15,000 g/mol, while the weight average molar mass was 29,500 g/mol, leading to a polydispersity index of 1.97 with a single molar mass distribution (data not shown). In addition, ^1^H NMR analysis was performed to characterize the apparent molar ratio of NIPAM to AAh in the random copolymer, poly(NIPAM-co-AAh), as well as the incorporation of methacrylic moieties in methacrylated poly(NIPAM-co-AAh) and PEGDMA. The feed molar ratio of NIPAM to AAh for the synthesis of poly(NIPAM-co-AAh) was 3:1, to introduce the methacrylic group into the AAh and have thermal responsiveness of the random copolymer at ~37°C. The apparent molar ratio of NIPAM to AAh of poly(NIPAM-co-AAh) was 2.74:1, suggesting that AAh monomer units were preferentially incorporated into the growing copolymer chains compared to the NIPAM monomers. The ^1^H NMR spectra of methacrylated poly(NIPAM-co-AAh) and PEGDMA in Figures 2A,B show that methacrylic moieties were incorporated into the allylamine residues of poly(NIPAM-co-AAh) and the hydroxyl groups of PEGs when they were separately incubated with MA and purified. The chemical shifts of the methylene protons of the two polymers in the ^1^H NMR spectra were ~5.7 ppm and ~6.2 ppm, confirming the presence of methacrylic groups. Incorporation efficiency of methacrylic groups into allylamine residues of poly(NIPAM-co-AAh) was calculated by measuring the peak areas of (d) C-H groups of poly(NIPAM) moieties and (e, f) methylene protons of methacrylated poly(AAh) moieties in Figure 2A. As the apparent molar ratio of NIPAM to either AAh of poly(NIPAM-co-AAh) or methacrylic group of methacrylated poly(NIPAM-co-AAh) was 2.74:1 or 7.14:1, incorporation efficiency of the methacrylic anhydride into poly(NIPAM-co-AAh) was 38.36 %. In addition, the degree of incorporation of methacrylic groups into hydroxyl groups of PEG was calculated by integrating the areas under the NMR peaks, corresponding to (b, c) methylene protons of PEGDMA and (e) the protons adjacent to the terminal methacrylic group, as shown in Figure 2B. As a result, the incorporation efficiency of the methacrylic groups to PEG was 51.16 % based on the ^1^H NMR analysis. Furthermore, the temperature responsiveness of both poly(NIPAM-co-AAh) and methacrylated poly(NIPAM-co-AAh) in aqueous solutions was characterized using UV-Vis spectrometry in the concentration range of 0.5–2.0 w/v%. Figure 2C shows the optical absorbance of poly(NIPAM-co-AAh) and methacrylated poly(NIPAM-co-AAh) as a function of temperature. The turbidity of poly(NIPAM-co-AAh) was dramatically increased in a narrow temperature range around 35–38 °C at 0.5 and 2.0 w/v% because of thermally-induced aggregation of the poly(NIPAM) copolymer chains. As the poly(NIPAM-co-AAh) concentration serially increased, the thermal transition temperature (T_t_) decreased due to enhanced entanglement of polymer chains at higher concentrations, as previously reported. Moreover, the T_t_ of methacrylated poly(NIPAM-co-AAh) decreased by ~4–5 °C compared to that of poly(NIPAM-co-AAh) at the identical polymer concentration, due to introduction of methacrylic moieties into the allylamine residues of poly(NIPAM-co-AAh) using MA.

### Nanoscale Characterization of SCNFs and BCNFs

**Figure 3:**
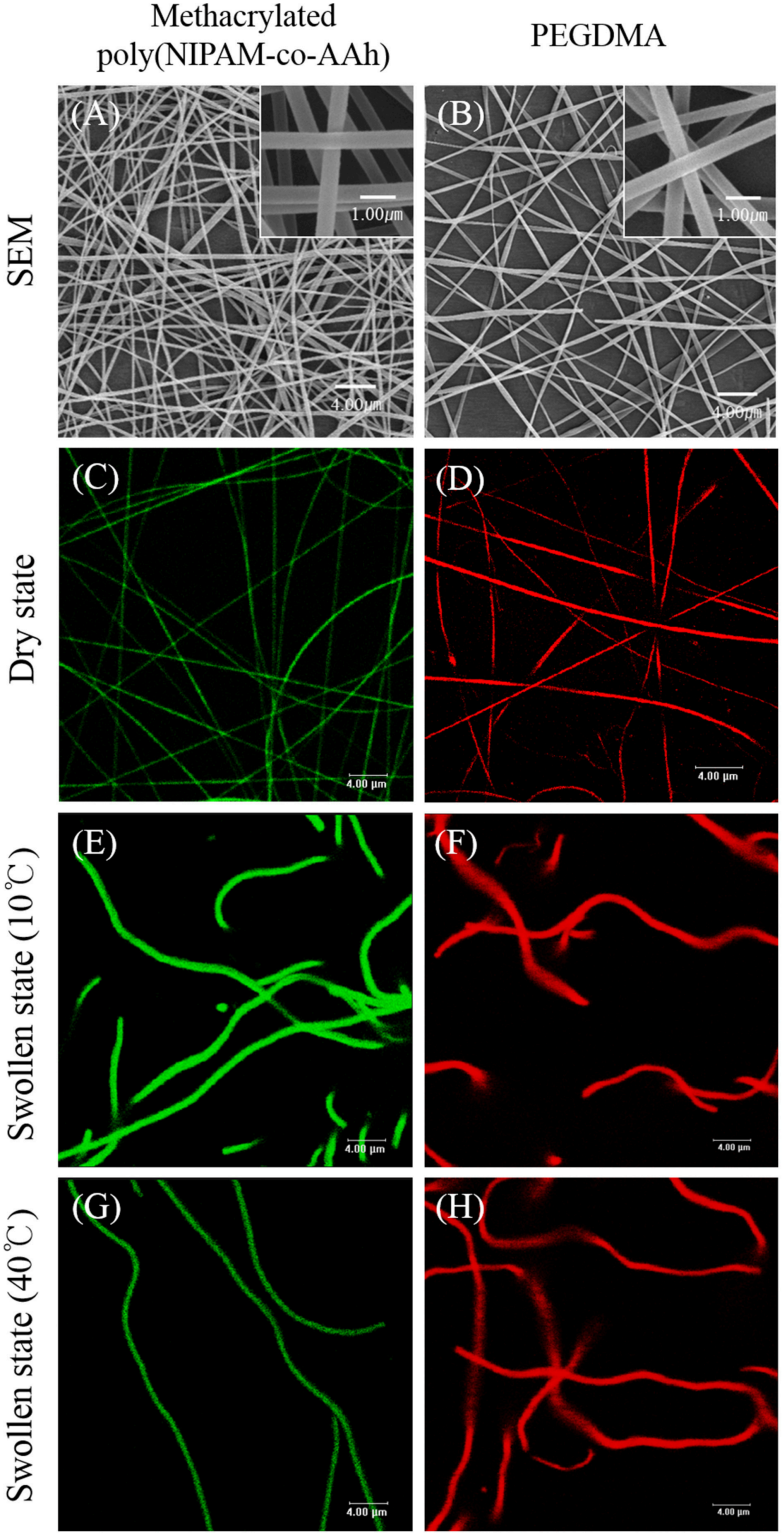
SEM images **(A,B)** and fluorescence images **(C–H)** of the single compartmental nanofibers of chemically crosslinked, methacrylated poly(NIPAM-co-AAh) **(A,C,E,G)** and PEGDMA **(B,D,F,H)** in the dry state **(A–D)** and swollen state **(E–H)** at 10°C **(E,F)** and 40°C **(G,H)**. Scale bars are 4.0 μm in **(A–H)** and 1.0 μm in the insets of **(A)** and **(B)**.

To synthesize the BCNFs with actuation at the nanoscale under aqueous conditions, the EHD jetting conditions for SCNFs composed of either methacrylated poly(NIPAM-co-AAh) with fluorescein diacetate or PEGDMA with Nile red, were optimized by adjusting both viscosity and flow rates of the polymer solutions as well as the voltage applied. Furthermore, their morphologies in dry and swollen states as well as their thermally-induced volume transitions were evaluated using SEM and CLSM imaging. Figure 3 shows SEM images (A, B) and fluorescence images (C–H) of the SCNFs of chemically crosslinked, methacrylated poly(NIPAM-co-AAh) (A, C, E, and G) and PEGDMA (B, D, F, and H). The SEM images of the SCNFs show that continuous nanofibers with uniform morphology were produced without any significant beads-on-a-string structures. As measured from the SEM images of five different batches (*N* = 5) for each SCNF, the average diameters with standard deviations of the SCNFs of methacrylated poly(NIPAM-co-AAh) and PEGDMA were 478 ± 201 nm and 383 ± 83 nm, respectively, which is in good agreement with the diameters of the CLSM images in a dry state, as shown in Figures 3C,D. The UV irradiation-mediated, solid phase crosslinking of both SCNFs in the presence of 2-hydroxy-2-methyl-propiophenone as the photoinitiator, was confirmed by immersing the materials in PBS at 10°C. As shown in Figures 3E,F, both UV-crosslinked SCNFs maintained their intrinsic nanofiber shape and morphology upon exposure to water, while increases in the size of both types of SCNFs were observed due to the hydrogel-like property of these hydrophilic polymer networks. This confirms that nanofibrous structures were successfully stabilized in dry and swollen states because of the UV-initiated chemical stabilization of methacrylated poly(NIPAM-co-AAh) and PEGDMA SCNFs. Figures 3E,G indicate that the average diameter of methacrylated poly(NIPAM-co-AAh) SCNFs in the swollen state significantly changed from 0.99 to 0.65 μm via volume change as the temperature increased from 10°C (below its LCST) to 40°C (above its LCST), because the methacrylated poly(NIPAM-co-AAh) chains instantaneously aggregated above the LCST. On the other hand, Figures 3F,H show that PEGDMA SCNFs with an average diameter of 1.04 μm in the swollen state showed no volume transition at 10 or 40°C due to lack of thermoresponsiveness and the stabilized hydrogel network structure of PEGDMA in PBS, which we attribute to UV-initiated chemical crosslinking.

**Figure 4:**
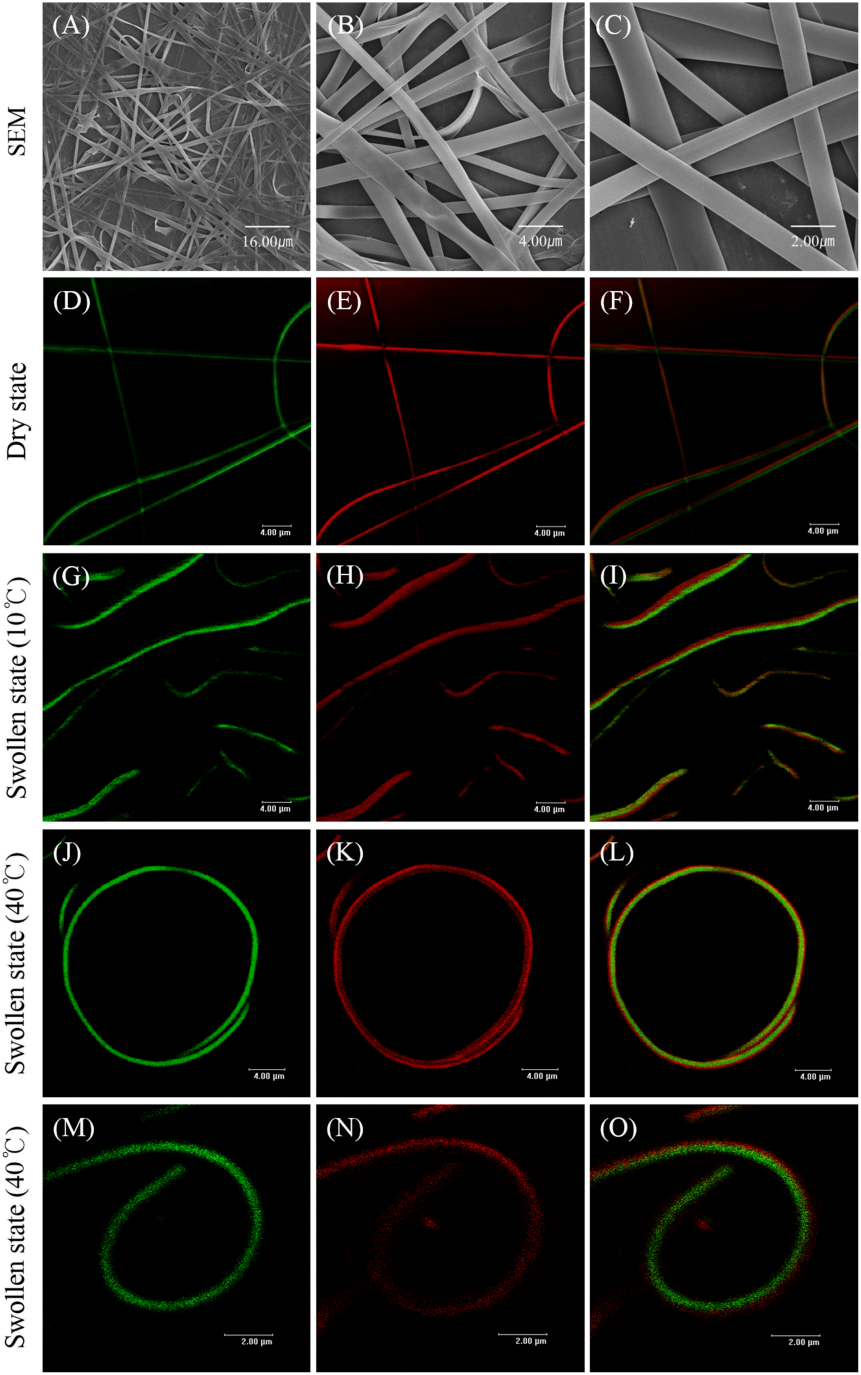
SEM images **(A–C)** and fluorescence images **(D–O)** of stimuli-responsive, chemically crosslinked bicompartmental nanofibers composed of thermoresponsive, methacrylated poly(NIPAM-co-AAh) and non-thermoresponsive PEGDMA in each compartment in the dry state **(A–F)** and swollen state **(G–O)** at 10°C **(G–I)** and 40°C **(J–O)**. Scale bars are 16.0 μm in **(A)**, 4.0 μm in **(B,D–L)**, and 2.0 μm in **(C,M–O)**.

With optimized EHD jetting conditions of the SCNFs composed of either polymer, the EHD co-jetting of the two polymer solutions of methacrylated poly(NIPAM-co-AAh) with fluorescein diacetate and PEGDMA with Nile red in an ethanol and TFE mixture at a 3:1 volume ratio was performed to synthesize the BCNFs composed of poly(NIPAM-co-AAh) and PEGDMA compartments. The SEM images of the BCNFs in the dry state, at different magnification ratios in Figures 4A–C, demonstrate that continuous nanofibers with uniform morphology were formed without formation of the beads-on-a-string structures, and their average diameter with standard deviation was 1.06 ± 0.78 μm, as determined by the SEM images of five different batches (*N* = 5) of the BCNFs. In contrast, their fluorescence images in Figures 4D–F clearly represent a distinct interface between two compartments, as confirmed by the separate fluorescence signals of fluorescein diacetate and Nile red from the two different compartments. When the BCNFs were immersed in PBS at pH 7.4 and 10°C, their average diameter changed from 1.06 to 1.94 μm, because the two compartments were swollen to similar degrees due to the chemically crosslinked, hydrophilic polymer networks, as observed in Figures 4G–I. Importantly, we noted that the two compartments retained a discrete interface in both dry and swollen states, indicating that there was no severe inter-diffusion between the two polymer solutions during EHD co-jetting, and that UV-initiated chemical stabilization had no significant effect on the degree of compartmentalization of the BCNFs even in the swollen state. Furthermore, CLSM images of different BCNFs in Figures 4J–O show that their average diameter in the swollen state changed from 1.94 to 1.35 μm, and that the BCNFs underwent a rapid transition from a linearly extended form to a coil-like one as the temperature increased from 10°C (below their LCST) to 40°C (above their LCST). This resulted from thermally-induced volume decrease of the poly(NIPAM-co-AAh) compartment because of the instantaneous collapse of the poly(NIPAM) copolymers above their LCST and maintenance of the original volume of the PEGDMA copolymer in the swollen state irrespective of temperature, which is in good agreement with a previous study (Jalani et al., 2014). Moreover, the thermal stimulus-triggered transitions of these chemically crosslinked BCNFs, between a fully stretched shape and a coiled one, were completely reversible and stable without any significant aggregation of the nanofibers under aqueous conditions.

### *In vitro* Drug Release of Dual Drug-Loaded BCNFs

**Figure 5:**
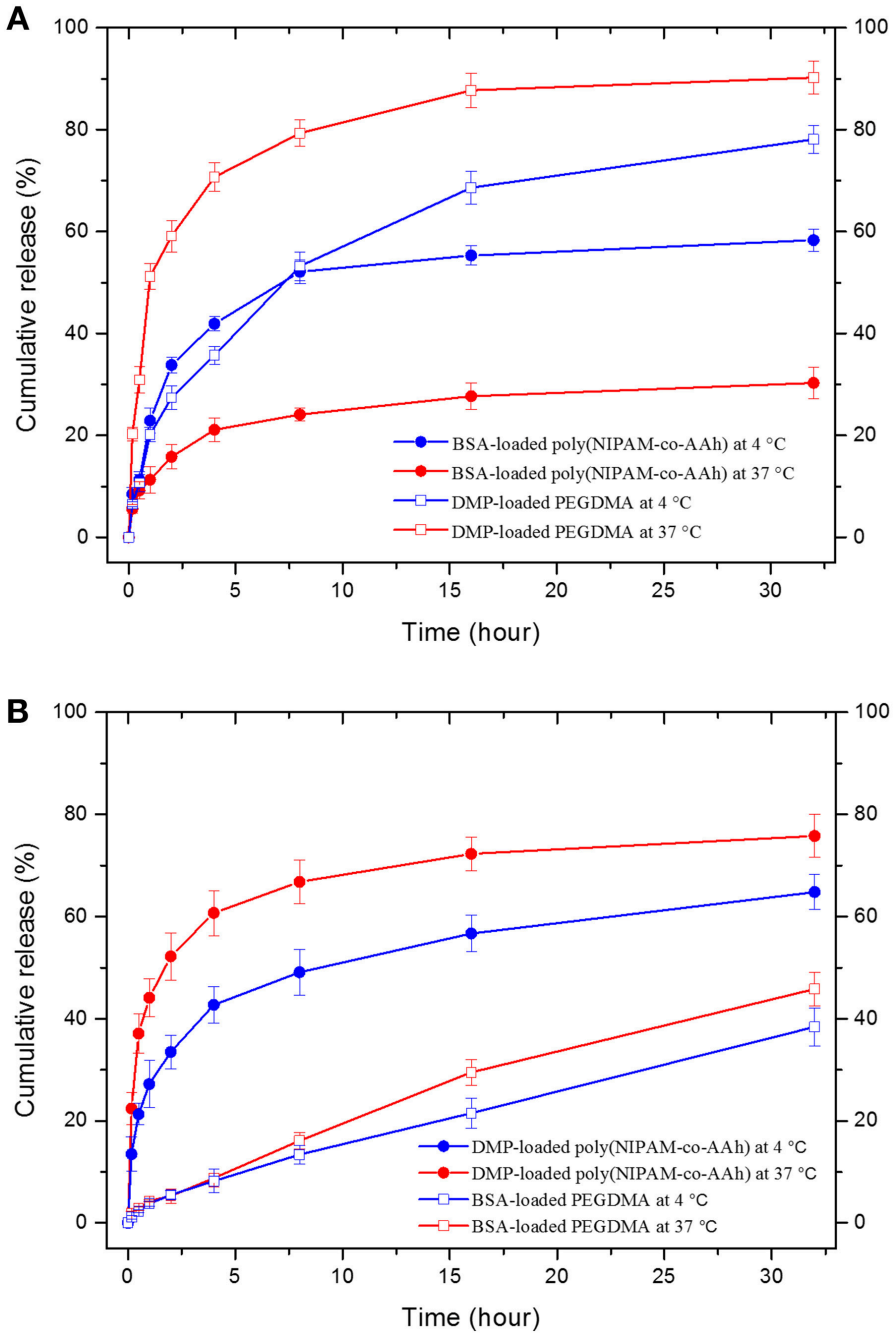
*In vitro* drug release profiles from two bicompartmental nanofibers (BCNFs), **(A)** BCNFs with a BSA-loaded poly(NIPAM-co-AAh) compartment and a DMP-loaded PEGDMA one and **(B)** BCNFs with a DMP-loaded poly(NIPAM-co-AAh) compartment and a BSA-loaded PEGDMA one at temperatures of 4°C (below lower critical solution temperature [LCST]) and 37°C (above LCST).

Finally, as shown in Figure 5, *in vitro* drug release studies of (A) BCNFs with a BSA-loaded poly(NIPAM-co-AAh) compartment and a DMP-loaded PEGDMA compartment and (B) BCNFs with a DMP-loaded poly(NIPAM-co-AAh) compartment and a BSA-loaded PEGDMA one were performed at two temperatures; 4°C (below LCST) and 37°C (above LCST). The release of BSA from the thermoresponsive poly(NIPAM-co-AAh) compartment at 37°C was slower than that at 4°C due to the increased entrapment of BSA as the biomacromolecule, because the hydrated pore size of the collapsed poly(NIPAM) copolymer networks decreased at 37°C via volume change. On the other hand, release of DMP from the non-thermoresponsive PEGDMA compartment at 37°C was faster compared to that at 4°C, potentially due to enhanced mobility and diffusion of small molecules at elevated temperature under the identical PEGDMA networks without any volume change. In contrast, release of BSA from the PEGDMA compartment at 4 and 37°C was very slow for 32 h and showed a sustained release behavior without any initial burst, because the BSA from the PEGDMA was mostly entrapped to a similar degree irrespective of temperature. However, DMP release from the poly(NIPAM) copolymer compartment at 37°C was faster than that at 4°C. This suggests that there was no significant effect of volume change of poly(NIPAM-co-AAh) on release of DMP because it is small enough to be released irrespective of pore size. In addition, to obtain the diffusion mechanism of DMP and BSA from two different compartments at 4 and 37°C, the data obtained from the drug release studies were fitted to a Korsmeyer-Peppas model, defined as M_t_/M_∞_ = Kt^n^, where M_t_/M_∞_ is a fraction of drug released at time t, K is a release rate constant, and n is a release exponent (Sohrabi et al., 2013; Sampath et al., 2014). The values of both release rate constant (K) and release exponent (n) were determined with regression coefficient (R^2^) to clarify whether the type of drug release mechanism follows Fickian diffusion (*n* ≤ 0.45) or anomalous (non-Fickian) diffusion (0.45 < *n* < 1) for the drug-loaded nanofibers. The first 60% of the release curve for a short-range time was fitted to Korsmeyer-Peppas model. Table S1 (Supporting Information) shows the calculated release exponent value (n) and regression coefficient (R^2^) of drug release from the respective compartment at different temperatures. The high R-squared values (R^2^ > 0.9) tabulated in Table S1 indicate that Korsmeyer-Peppas model used in this study fits the drug release data with close approximation. Fickian diffusion was the primary release mechanism for the poly(NIPAM-co-AAh) compartment, while non-Fickian diffusion-controlled release was observed for the PEGDMA compartment. The non-Fickian diffusion of DMP and BSA from the PEGDMA compartment occurred through the combined effect of diffusion and surface erosion of the PEGDMA network, which would be potentially due to low degree of chemical crosslinking of PEGDMA.

## Conclusion

We prepared thermally-responsive, chemically crosslinked BCNFs with reversible nanoscale actuation by EHD co-jetting with side-by-side needle geometry for decoupled drug release. Two photocrosslinkable polymers, methacrylated poly(NIPAM-co-AAh), and PEGDMA, in distinct compartments of the BCNFs, were stabilized in the solid state via UV irradiation, and these BCNFs in their swollen state maintained a clear interface under physiological conditions. Their reversible actuation induced by a temperature trigger of thermally-induced volume change of the chemically crosslinked poly(NIPAM-co-AAh) compartment and the identical volume of the non-thermoresponsive PEGDMA one. Importantly, temperature-dependent, decoupled drug release profiles from the two different, chemically crosslinked polymer compartments of these BCNFs, with precisely engineered nanoscale compartmentalization, can be potentially useful for temporally multi-modal drug delivery reservoirs and functional nanofibers with actuation at the nanoscale, for tissue engineering and regenerative medicine. Future work should focus on fabrication of directionally aligned BCNFs, which would have great potential for temperature-responsive bending hydrogels as actuating cell sheets with oriented cell growth via spatio-selective cell-surface interactions and also as soft actuator-based robotic systems (Stoychev et al., 2013; Wang et al., 2013; Stoychev and Ionov, 2016; Hessberger et al., 2018).

## Author Contributions

DL, CJ, JL, and GJ contributed to design and synthesis of polymers and bicompartmental nanofibers (BCNFs) and data analysis; CJ, JL, and GJ performed characterization of polymer and BCNFs, and wrote each section of the first draft; EH revised the draft. DL contributed to critical revision based on actuation mechanisms of the BCNFs at nanoscale.

### Conflict of Interest Statement

The authors declare that the research was conducted in the absence of any commercial or financial relationships that could be construed as a potential conflict of interest.
