# Supplementary material for: Thermally-Induced Actuations of Stimuli-Responsive, Bicompartmental Nanofibers for Decoupled Drug Release
Source: Front Chem. 2019 Feb 19;7:73. doi: 10.3389/fchem.2019.00073 (PMC6390475; doi:10.3389/fchem.2019.00073)
Supplement: Supplementary file 1 [file Table_1.docx]

**Electronic Supporting Information (ESI)**

**Thermally-induced Actuations of Stimuli-responsive, Bicompartmental Nanofibers for Decoupled Drug Release**

Chan Woo Jung^⊥^, Jae Sang Lee^⊥^, Ghulam Jalani, Eun Young Hwang, and Dong Woo Lim*

Department of Bionano Engineering and Bionanotechnology, College of Engineering Sciences, Hanyang University, Ansan, South Korea

^⊥^ These authors contributed equally to this work.

* Corresponding author. Department of Bionano Engineering and Bionanotechnology, College of Engineering Sciences, Hanyang University, Ansan, Republic of Korea; E-mail: [dlim@hanyang.ac.kr](mailto:dlim@hanyang.ac.kr)

**Methods**

**Diffusion mechanism of drug release from the respective compartments of the BCNFs**

The data obtained from the drug release studies in Figure 5 were fitted with Korsmeyer-Peppas model to obtain the diffusion mechanism of DMP and BSA from two different compartments at 4 °C and 37 °C. This model is defined as M_t_/M_∞_ = Kt^n^, where M_t_/M_∞_ is a fraction of drug released at time t, K is a release rate constant, and n is a release exponent (Sohrabi et al., 2013;Sampath et al., 2014). The values of both release rate constant (K) and release exponent (n) were determined with regression coefficient (R^2^) to clarify whether the type of drug release mechanism follows Fickian diffusion (n ≤ 0.45) or anomalous (non-Fickian) diffusion ( 0.45 < n < 1) for cylindrical shaped matrices. The first 60% of the release curve was fitted to Korsmeyer-Peppas model. Table S1 (Supporting Information) shows the calculated release exponent value (n) and regression coefficient (R^2^) of drug release from the respective compartment at different temperatures.

**Table S1.** The calculated values of the release exponent (n) and regression coefficient (R^2^) of drug release from the respective compartments of the BCNFs at different temperatures.

| **Drug-loaded compartment of the BCNFs** | **R^2^** | **n** | **Release mechanism** |
| --- | --- | --- | --- |
| BSA-loaded poly(NIPAM-co-AAh) at 4 °C | 0.9074 | 0.5839 | Non-Fickian diffusion |
| BSA-loaded poly(NIPAM-co-AAh) at 37 °C | 0.9909 | 0.4118 | Fickian diffusion |
| DMP-loaded PEGDMA at 4 °C | 0.9736 | 0.5642 | Non-Fickian diffusion |
| DMP-loaded PEGDMA at 37 °C | 0.9374 | 0.5106 | Non-Fickian diffusion |
| DMP-loaded poly(NIPAM-co-AAh) at 4 °C | 0.9907 | 0.3706 | Fickian diffusion |
| DMP-loaded poly(NIPAM-co-AAh) at 37 °C | 0.9550 | 0.3897 | Fickian diffusion |
| BSA-loaded PEGDMA at 4 °C | *N/A | *N/A | *N/A |
| BSA-loaded PEGDMA at 37 °C | *N/A | *N/A | *N/A |

*N/A: Not available

**References**

Sohrabi, A., Shaibani, P., Etayash, H., Kaur, K., and Thundat, T. (2013). Sustained drug release and antibacterial activity of ampicillin incorporated poly (methyl methacrylate)–nylon6 core/shell nanofibers. *Polymer* 54**,** 2699-2705.

Sampath, M., Lakra, R., Korrapati, P., and Sengottuvelan, B. (2014). Curcumin loaded poly (lactic-co-glycolic) acid nanofiber for the treatment of carcinoma. Colloids and Surfaces B: Biointerfaces 117, 128-134.
